# Biological Evaluation of Polyvinyl Alcohol Hydrogels Enriched by Hyaluronic Acid and Hydroxyapatite

**DOI:** 10.3390/ijms21165719

**Published:** 2020-08-10

**Authors:** Petra Chocholata, Vlastimil Kulda, Jana Dvorakova, Jana Kolaja Dobra, Vaclav Babuska

**Affiliations:** Department of Medical Chemistry and Biochemistry, Faculty of Medicine in Pilsen, Charles University, Karlovarska 48, 301 66 Plzen, Czech Republic; petra.chocholata@lfp.cuni.cz (P.C.); vlastimil.kulda@lfp.cuni.cz (V.K.); jana.dvorakova@lfp.cuni.cz (J.D.); jana.dobra@lfp.cuni.cz (J.K.D.)

**Keywords:** scaffold, polyvinyl alcohol, hyaluronic acid, hydroxyapatite, bone tissue engineering, biological evaluation

## Abstract

This study aimed to develop polyvinyl alcohol (PVA) -based scaffold enriched with hyaluronic acid (HA) and hydroxyapatite (HAp) using physical crosslinking by freezing–thawing method. We accomplished biological evaluation of scaffolds, swelling degree, bioactivity assessment, and hemolytic test. The results showed that all types of scaffolds should be safe for use in the human body. The culturing of human osteoblast-like cells MG-63 and their proliferation showed better adhesion of cells due to the presence of HA and confirmed better proliferation depending on the amount of HAp. This paper gives the optimal composition of the scaffold and the optimal amount of the particular components of the scaffold. Based on our results we concluded that the best PVA/HA/HAp combination is in the ratio 3:1:2.

## 1. Introduction

Bone transplantation is the second most common type of tissue transplantation following blood transfusion [1]. Bone grafting is considered as a well-described technique of bone defect treatment [2]. Bone grafting refers to the transplantation of an autograph or an allograft. Despite both transplantation types meeting the requirements of appropriate properties (osteoconductivity, osteoinductivity, osteointegration), there are many disadvantages, e.g., low availability of grafts, blood loss, longer surgical time, infection etc. Bone tissue engineering tries to eliminate these problems by the application of fully biocompatible scaffolds based on natural or synthetic materials serving as matrices for cell incorporation and cultivation support for renewal of healthy tissue [1,2].

The main objective of tissue engineering is to restore and improve the function of tissues by preparing porous three-dimensional (3D) scaffolds, and seeding them with cells and growth factors [3]. The term scaffold is used for 3D biomaterial that provides a suitable environment to promote cell proliferation, osteogenic differentiation, and production of extracellular matrix (ECM) in order to regenerate tissues and organs. Currently, there is the aim to produce scaffolds able to provide regenerative signals to cells [4]. For this purpose, efforts are being made to develop scaffolds based on biomaterials that mimic those found in the natural environment [1]. Attention must be paid to the design and composition of the desired scaffolds [5]. Different types of materials can be currently used for bone tissue scaffold fabrication. There are efforts to obtain hybrid scaffolds with proper characteristics by using combinations of natural and synthetic polymers in combination with inorganic materials in the form of hydrogel [2]. A hydrogel is a three-dimensional unique, soft, and hydrophilic biomaterial [6], composed of a polymeric network that is able to tightly bind large quantities of water without dissolving [7]. Due to the high content of water, hydrogels show a flexibility similar to natural tissue. The objective of the presented study was to produce a hydrogel that includes both synthetic and natural as well as inorganic components and thereby to balance the advantages and disadvantages of this scaffold for use in bone tissue engineering. Therefore, the hydrogel scaffold based on polyvinyl alcohol as a synthetic and hyaluronic acid as a natural component, supplemented with hydroxyapatite, was chosen for this study.

Polyvinyl alcohol (PVA) is an often tested synthetic polymer with a good biocompatibility [8]. It can be transformed into hydrogel easily through the use of repeated freeze-thaw cycles [9]. This physical crosslinking, based on the reaction of side hydroxyl groups of PVA [10], is very convenient as there is no need to use any chemical cross-linker that may cause toxicity [9]. It was found that the number of freeze-thaw cycles, freezing temperature, time, and the concentration of PVA affect structure and the resulting physical [11] and mechanical properties [12]. The higher the number of freeze-thaw cycles, the higher the stiffness of the polymer and the loss of PVA chains orientation was observed [13]. A high hydrophilicity of PVA hydrogels causes suppression of cell adherence to it. However, its intrinsic cell-non-adhesion provides poor support to cell growth and integration to peripheral tissues. PVA could be blended with natural macromolecules, such as chitosan, starch, gelatin, hyaluronic acid, and so on [10]. Modification of PVA has shown improvements in cell adhesion and growth, for example by hydroxyapatite (HAp). The incorporation of HAp into PVA hydrogel enhanced cell density with good cell spreading morphology.

Hydroxyapatite (HAp), Ca_10_(PO_4_)_6_(OH)_2_, as a major natural inorganic component of bone, shows excellent bioactivity, biocompatibility, osteoconductivity, non-toxicity, and non-inflammatory characteristics [1]. Its mechanical properties are essentially influenced by the size of the HAp particles, porosity, density, etc. [14]. HAp is very hard but brittle, with a very slow degradation rate in vivo, and that is why it should be joined with natural or synthetic polymers to create scaffolds. On the other hand, HAp is very beneficial for constructing bones, because it promotes the adhesion and proliferation of osteoblasts cultured in vitro [15]. It stimulates growth factors (e.g., bone morphogenic protein) and elevates activity of alkaline phosphatase (ALP) in mesenchymal stem cells (MSCs) [1].

Hyaluronic acid (HA) is abundant throughout the ECM, especially connective tissues, and as a structural molecule [5] in the human body. It is a natural polysaccharide composed of a linear glucosaminoglycan, where repeated units of N-acetyl-D-glucosamine and D-glucuronic acid are linked by alternating β-1,3- and β-1,4- glycosidic bonds [16]. Not only its biocompatibility and biodegradability but also its viscoelasticity are convenient properties for using HA in biomedicine, health care, and cosmetics. A very significant advantage of HA is its enzymatic degradability by hyaluronidase, an enzyme produced by mammalian cells. In view of the very rapid degradation and water solubility of HA, it is advisable to cross-link it or to blend it with another natural or synthetic polymer [1].

The aim of our study was to produce a scaffold based on PVA in combination with HA and enriched with HAp and to examine the effect of scaffold composition on the adhesion and proliferation of human osteoblast-like cell line MG-63 cultured in static conditions. According to our knowledge, this combination of all three materials PVA, HA and HAp together was new.

## 2. Results and Discussion

### 2.1. Swelling Degree

The swelling degree is one of the basic parameters for scaffold application in tissue engineering. The composition and the hydrophilic nature influence the swelling degree. The absorption of water is related to pore size and pore interconnection [17]. The swelling degree was estimated as an average value (Table 1) and the results confirmed that both HAp and even HA influenced the swelling behavior of scaffolds. The same types of scaffolds but with different amounts of HAp showed a decrease in swelling degree with an increase in the amount of HAp. The higher the concentration of HAp in the sample, the lower the swelling degree observed due to the lower hydrophilicity, as seen in samples HB1 and HB3 (*p* < 0.001), HB2 and HB3 (*p* = 0.0107), and HB1 and HB2 (*p* = 0.0306). HAp made the composites stiffer, decreasing the degree of swelling [18].

Compared to HAp, HA increased the hydrophilicity and so the absorption of water. The samples containing the higher proportion of HA and the same amount of HAp showed a higher swelling degree, as evidenced by samples HA3 and HB3 (*p* = 0.0113). Kaur et al. evaluated the swelling behavior of scaffolds containing only PVA and HAp, without HA [18]. The degree of swelling also decreased with the increase of HAp concentration but our total swelling degree was much higher due to adding the hydrophilic component, namely HA.

### 2.2. Bioactivity Assessment

The samples of each scaffold type were used for the evaluation of bioactivity and the weight increase was determined as an average value. It was stated that a necessary requirement for the binding of the material to the bone tissue is the apatite-like crystal formation on its surface. The formation of apatite-like crystals in vivo could be reproduced in simulated body fluid (SBF). The material could be considered bioactive in vivo, if apatite crystals are formed on its surface in SBF [20]. Recently, the method for in vitro testing the scaffolds immersed into SBF has been standardized, although some authors disagree with this method. The reason for this is that there are some bioactive materials that do not form apatite crystals on their surface in SBF [21]. The apatite-like crystal formation in SBF should be supported by presence of HA [5]. We can confirm the weight increase of all scaffolds after 25 days (Table 1). The concentration of HA had a direct correlation with the mass increase, as evidenced by samples A and B (*p* = 0.0289). The presence of HA influenced the bioactivity of samples due to the increase of hydrophilicity. It was found that the hydrophilic surface is more bioactive [5]. The crystal formation is probably enabled due to the interaction between HA in the scaffold and water-soluble ions [5]. We observed a higher mass in samples containing a higher amount of HA, as evidenced by samples A and B (*p* = 0.0289). There was no significant effect of the amount of HAp in the scaffolds on weight growth, i.e., on scaffold bioactivity. Kaur et al. performed a bioactivity assessment of scaffolds composed of PVA and HAp [18]. The results showed weight loss dependent on HAp concentration; the higher the HAp concentration, the lower the weight loss. Nikbakht et al. evaluated the bioactivity assessment of scaffolds composed of HA on poly (3-hydroxybutyrate) [5]. The results illustrated the direct correlation of HA concentration and crystal growth.

### 2.3. Hemolytic Test

It is important to study interaction of scaffold materials and blood components because of a blood clotting risk. The test was based on the determination of the amount of hemoglobin released from red blood cells when blood was in contact with scaffolds [19]. All determined results, as average values (Table 1), had values between 0.1% and 1.55%. All types of samples produced by us were highly compatible with human blood in respect of all components of scaffolds, i.e., they are biocompatible. If we compared the samples that differed only in quantity of HAp, there was a direct dependence. The hemolysis increased with the higher amount of HAp. It was observed that there was increased hemolysis of samples with an amount of added HAp in the ratio PVA/HAp 1:1 (1.55%, 1.46%) compared with samples where no HAp was added (0.97%, 0.68%) and where a lower ratio of PVA/HAp (3:1, 3:2) (0.39%, 1.46%, 0.10%, 0.58%) was present. The increased hemolysis for samples with a higher amount of HAp meant a decrease of blood compatibility. This can be caused by the interaction between ionic groups of HAp and blood which cause higher hemolysis [18]. It is necessary to say that it was only a preliminary test and an extended examination of the biocompatibility must be done. Compared to the results of Kaur et al. and Pal et al. [18,19], it can be said that adding HAp did not influence hemocompatibility and that all types of scaffolds were highly hemocompatible.

### 2.4. Cell Viability

**Figure 1:**
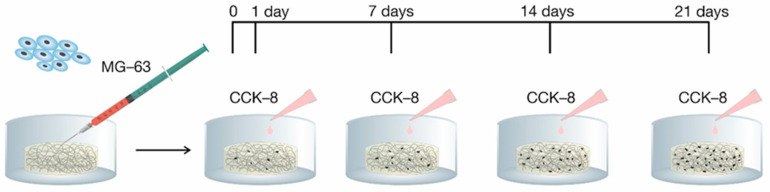
Timeline of adhesion and proliferation monitoring. CCK-8 is Cell Counting Kit-8.

Cell adhesion and proliferation is an important factor for integration of a scaffold into a biological environment. The in vitro evaluation was accomplished through the use of human osteoblast-like cells MG-63. The cell adhesion (24 h after seeding cells) and proliferation (in day 7, 14, and 21 after seeding cells) were estimated by using Cell Counting Kit-8 (CCK-8) (Figure 1).

**Figure 2:**
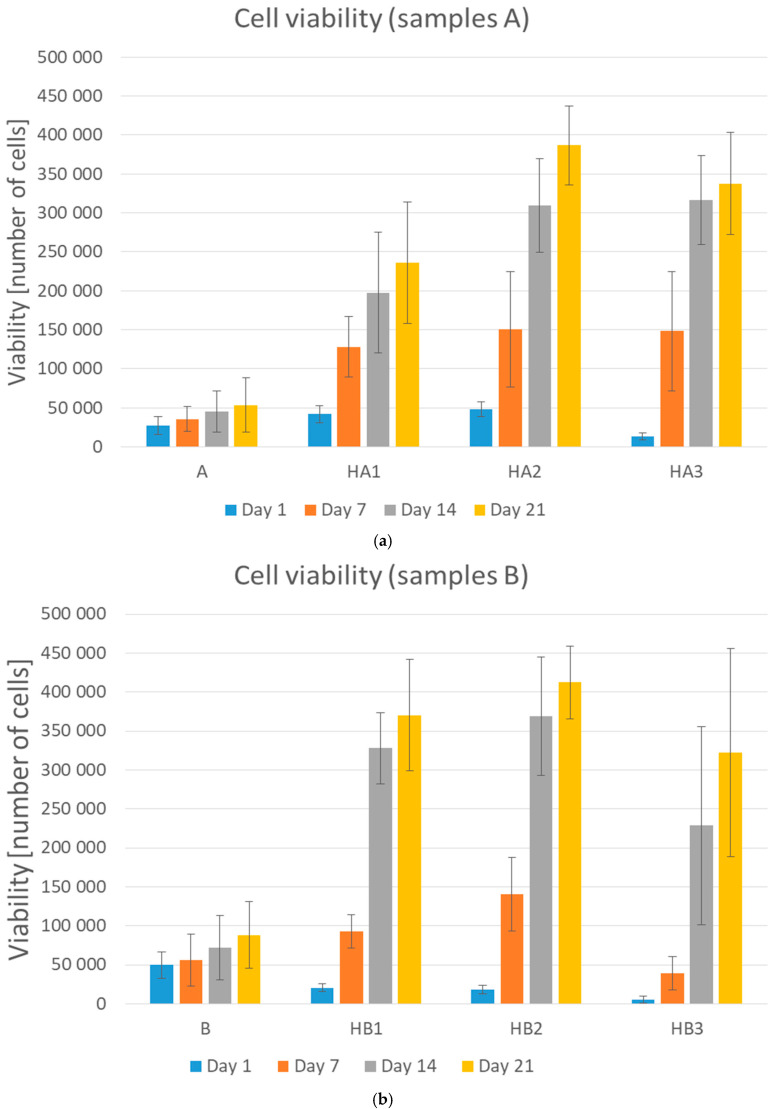
Graphical representation of cell viability (adhesion—day 1, proliferation—day 7, 14, and 21). (**a**) Samples A, HA1, HA2, and HA3 and (**b**) samples B, HB1, HB2, and HB3. Error bars represent ±SD. A: PVA/HA = 3:1; HA1: PVA/HA/Hap = 3:1:1; HA2: PVA/HA/Hap = 3:1:2; HA3: PVA/HA/Hap = 3:1:3; B: PVA/HA = 1:1; HB1: PVA/HA/Hap = 1:1:1; HB2: PVA/HA/Hap = 1:1:2; HB3: PVA/HA/Hap = 1:1:3.

During the cell culturing, the absorption value of formazan increased which suggested cell proliferation on the scaffolds. The primary adhesion measured 24 h after cell seeding on PVA/HA/HAp scaffolds increased compared to the primary adhesion on the PVA hydrogel in our preliminary study. The scaffolds prepared just from PVA indicated insufficient adhesion of cells, which became evident mostly during the proliferation and during the operation with hydrogels. A higher amount of HA, meant that higher primary adhesion and proliferation were observed, especially after longer term culturing. It indicated that HA made it possible for cells to attach more strongly, probably due to the natural biological functions of HA. These results confirmed the findings of Oh et al. [11], who also showed the same effect of HA on cell adhesion and proliferation. The addition of HAp improved cell spreading and cell density on the surface area in comparison with PVA/HA matrix without any Hap; samples A and HA1 (*p* = 0.0326), A and HA2 (*p* < 0.001), A and HA3 (*p* < 0.001) (Figure 2a), B and HB1 (*p* < 0.001), B and HB2 (*p* < 0.001) (Figure 2b).

**Figure 3:**
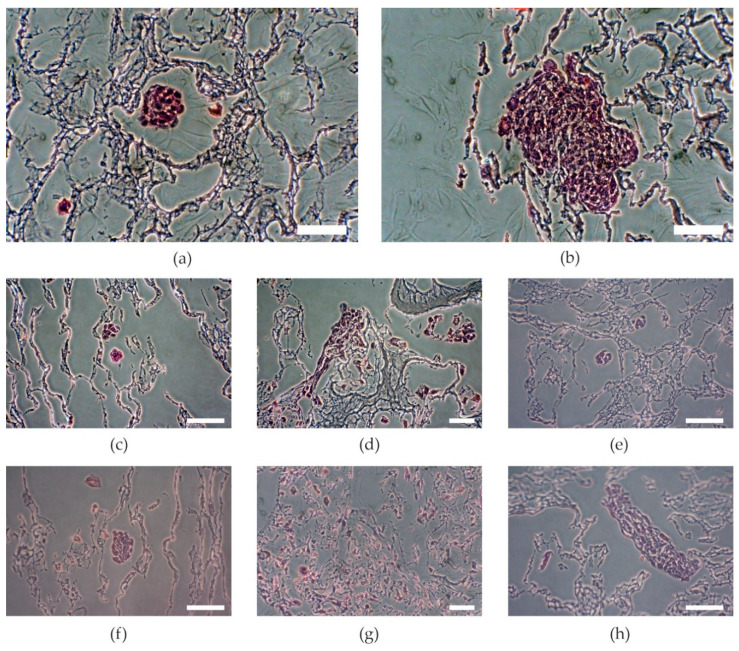
Visual comparison of MG-63 cell colonies stained with hematoxylin and eosin. (**a**) Sample A, (**b**) sample B, (**c**) sample HA1, (**d**) sample HA2, (**e**) sample HA3, (**f**) sample HB1, (**g**) sample HB2, (**h**) sample HB3. A: PVA/HA = 3:1; HA1: PVA/HA/Hap = 3:1:1; HA2: PVA/HA/Hap = 3:1:2; HA3: PVA/HA/Hap = 3:1:3; B: PVA/HA = 1:1; HB1: PVA/HA/Hap = 1:1:1; HB2: PVA/HA/Hap = 1:1:2; HB3: PVA/HA/Hap = 1:1:3. Scale bar = 50 µm.

This could be probably due to the higher surface area. The higher the concentration of HAp, the better the proliferation observed, as per samples HA1 and HA2 (*p* = 0.0159), but further increasing the amount of HAp decreased the proliferation, as per samples HB2 and HB3 (*p* < 0.001). The samples with the lowest amount of HAp showed the lowest increase of proliferation. The proliferation increased with increasing amounts of HAp, but the samples with the highest amount of HAp showed a decrease of proliferation (Figure 2) as Kaur et al. had noted in their results of cell adhesion and proliferation on PVA/HAp scaffolds [18]. On the other hand, higher amounts of HAp decreased primary adhesion. The higher the concentration of HAp, the lower was the primary adhesion of cells (Figure 2, samples HA3 and HB3) as an effect of increasing crystallinity and contact angle [18]. These results show that an optimal concentration of Hap is needed. Of the three tested concentrations, the mixtures in ratio HA/HAp 1:2 appeared to be optimal. Visual comparison of proliferation in particular samples was assessed through histological stained slices (Figure 3), which show different amounts and attachment of cells.

## 3. Materials and Methods

### 3.1. Materials

Polyvinyl alcohol (PVA, Mw 145,000, fully hydrolyzed), Ca(NO_3_)_2_·4H_2_O, NaCl, NaHCO_3_, KCl, K_2_HPO_4_·3H_2_O, MgCl_2_·6H_2_O (Merck, Prague, Czech Republic). Hyaluronic acid (HA, MW 1,800,000, ZVC Dr. Hoffmann, Citov pod Ripem, Czech Republic). KH_2_PO_4_ (Lach-Ner, Neratovice, Czech Republic). HCl, CaCl_2_, Na_2_SO_4_, Tris (Lachema, Brno, Czech Republic).

### 3.2. Synthesis of Hydroxyapatite

Hydroxyapatite (HAp, Ca_10_(PO_4_)_6_(OH)_2_, Ca/*p* = 1.67) was synthesized by the sol-gel method described elsewhere [22]. Briefly, KH_2_PO_4_ (0.6M) was dissolved in deionized water with stirring at room temperature. Ca(NO_3_)_2_ was added in amounts to keep the ratio Ca/*p* = 1.67. NH_3_ was used for retaining pH 10. The mixture was stirred for one hour and then it was left to mature for 24 h at room temperature. NH_3_ was removed by washing (up to neutral pH). The slurry of hydroxyapatite was dried for 48 h at 70 °C in the oven.

### 3.3. Preparation of Scaffolds

Aqueous solution of PVA (5%) was prepared by dissolving PVA powder in deionized water with stirring at 90 °C until a homogenous solution was obtained. Aqueous solution of HA (1%), was prepared by dissolving HA in deionized water with stirring at 50 °C until a homogenous solution was obtained. The mixtures of PVA and HA were prepared in a ratio of 75/25 (labelled as A), and 50/50 (labelled as B), stirred and slightly heated (around 40 °C). Aqueous solution of HAp (5%) was added to these two types of mixtures in ratio HA/HAp 1:1, 1:2, 1:3 (labelled HA1, HA2, HA3, HB1, HB2, HB3) and mixed properly. The mixtures were poured into 24-well plates and immediately frozen at −20 °C overnight. The final hydrogel was obtained by thawing a frozen solution at room temperature for 12 h (1 cycle) and this procedure was repeated for another 6 times, giving 7 cycles in total. Cylindrical hydrogel samples with a diameter of 1.5 cm were cut to a thickness about 5 mm. Samples were sterilized by immersion into 70% ethanol for 2 h. After the sterilization, scaffolds were washed in phosphate buffered saline (PBS) and treated in culture medium at 37 °C under 5% CO_2_ in a humidified incubator overnight to promote protein adsorption [13].

### 3.4. In Vitro Biological Evaluation of Scaffíolds

#### 3.4.1. Swelling Degree

The degree of swelling was estimated by soaking of freeze-dried samples into 10 mL of PBS solution at 37 °C and weighted up to invariable weight. Three samples of each scaffold type were used for determination of swelling degree (S_W_).

S_W_ [%] = [(m_i_ − m_f_)/m_i_] × 100 (1)

where m_i_ is the initial weight of the sample and m_f_ is the final invariable weight of the sample.

#### 3.4.2. Bioactivity Assessment

The bioactivity assessment of scaffolds was estimated as their ability to form calcium phosphate crystals on their surface during the incubation in SBF. According to prior research, HA supports the formation of calcium phosphate crystals [5]. The formation of apatite-like crystals was evaluated as a mass increase of scaffolds. SBF was prepared according to Kokubo protocol [5] (Appendix A).

The freeze-dried and weighed samples were immersed into the tubes with 10 mL of SBF, sealed and incubated in 37 °C for 25 days. After that, scaffolds were washed with deionized water and dried in a laboratory oven at 50 °C for 4 days. All samples were weighed and the weight increase was calculated using the following equation.

Weight increase [%] = [(m_a_ − m_b_)/m_b_] × 100 (2)

where m_b_ is the weight of the freeze-dried sample and m_a_ is the weight of the sample after incubation and drying.

#### 3.4.3. Hemolytic Test

Estimation of hemocompatibility of the composites by hemolytic test was performed according to Pal [19]. Fresh human blood (8 mL; in a test tube with sodium citrate) was diluted with 10 mL of physiological solution (0.9% NaCl). Tubes with 10 mL of physiological solution were pre-heated to 37 °C for 30 min. One sample was added to each tube, 0.2 mL of diluted blood was added to each sample and heated to 37 °C for 60 min. Three discoid replicates of each type of the scaffold were measured. As a negative control, a solution of 0.2 mL diluted blood in 10 mL of physiological solution was used. As a positive control, a solution of 0.2 mL of diluted blood in 10 mL of distilled water was used. All test tubes were heated to 37 °C for 60 min. After that all tubes were centrifuged for 5 min at 3000 rpm and 1 mL of supernatant was measured in cuvettes photometrically (UV/VIS Spectrophotometer Optizen POP Nano Bio, Mecasys Co., Daejeon, Korea) at 545 nm wavelength. The percentage of hemolysis was calculated for the estimation of hemocompatibility.

Hemolysis [%] = [(A_S_ − A_NC_)/(A_PC_ − A_NC_)] × 100 (3)

where A_S_ is the absorbance of the sample, A_NC_ is the absorbance of negative control and A_PC_ is the absorbance of positive control.

### 3.5. Cell Viability Tests

#### 3.5.1. Cell Cultures

Human osteoblast-like MG-63 cell line (ECACC 86051601, Sigma Aldrich, St. Louis, MO, USA), obtained from an osteosarcoma of a 14 year old male, was cultivated in Dulbecco’s Modified Eagle’s Medium (DMEM, Biosera Europe, Nuaille, France) supplemented with 10% (*v*/*v*) fetal bovine serum (FBS, Biosera Europe, Nuaille, France), 100 U/mL penicillin, 100 mg/mL streptomycin (PAA Laboratories GmbH, Pasching, Austria), and 2.5 mM stable glutamine (Diagnovum GmbH, Ebsdorfergrund, Germany), at 37 °C under 5% CO_2_ in a humidified incubator. Culture medium was refreshed as needed [23,24].

#### 3.5.2. Tests of Cell Adhesion and Proliferation

The samples were placed into 6-well plates (TPP Techno Plastic Products, Trasadingen, Switzerland), and 1 mL of the suspension with 4 × 10^5^ cells was seeded onto the scaffold using a syringe with a needle of 0.6 mm diameter. After 24 h, an initial adhesion was determined by using Cell Counting Kit-8 (CCK-8, Sigma Aldrich, Darmstadt, Germany) according to the protocol. The CCK-8 assay is based on the conversion of light purple highly water-soluble tetrazolium salt WST-8 [2-2-methoxy-4-nitrophenyl)-3-(4-nitrophenyl)-5-(2,4-disulfophenyl)-2H-tetrazolium, monosodium salt] to orange water-soluble formazan dye, which can be spectrophotometrically quantified. The amount of the formazan dye generated by the activity of dehydrogenases in cells is directly proportional to the number of living cells. Briefly, the samples were moved to a 24-well plate and 550 µL of premix (CCK-8 + DMEM) was added to each sample. After incubation (60 min, 37 °C, 5% CO_2_) samples were removed and the amount of formazan was determined photometrically at 450 nm (microplate reader SYNERGY H1, BioTek, Winooski, VT, USA). The number of viable cells was estimated based on a calibration curve. The primary adhesion was calculated according to the following formula.

Adhesion [%] = (N/N_0_) × 100 (4)

where N is the number of viable cells after 24 h post seeding and N_0_ is the number of seeded cells (400 × 10^3^). Cell proliferation was quantified as the number of viable cells based on a calibration curve by using CCK-8, on time points day 7, 14, and 21 post-seeding (Figure 1).

### 3.6. Histological Preparation

The samples were fixed and stored in 10% neutral-buffered formalin after day 21 of cell culturing. Standard dehydration in ethanol was performed followed by immersion in xylene, paraffin saturated xylene, and finally molten paraffin. Tissue blocks were cut at 5 μm (Microtom Leica RM2255, Leica Biosystems, Wetzlar, Germany) and stained by hematoxylin and eosin solutions (H&E) for cell visualization [25]. The stained slices were observed under an inverted optic microscope with a digital camera (Olympus CKX41, Olympus, Tokyo, Japan).

## 4. Conclusions

We reported fabrication of hydrogel based on mixtures of PVA and HA enriched by HAp using a freezing-thawing method of physical crosslinking. All samples showed the high hemocompatibility and the high swelling degree that is important for hydrogels intended to be used in tissue engineering. In summary, HA significantly increased the primary adhesion of cells and HAp improved the cell spreading and proliferation but only to a certain extent. A further increase in HAp content caused a decrease in cell proliferation. Based on our results we can conclude that the optimal composition of PVA/HA/HAp hydrogel was in the ratio 3:1:2 (sample HA2).

## Figures and Tables

**Table 1:** Results of swelling degree, bioactivity assessment, and the test of hemocompatibility.

Sample	Composition Ratio [PVA/HA/HAp]	Swelling Degree [%]	Bioactivity Assessment [%]	Hemolysis [%] ^1^
A	3:1:0	902.06	110.55	0.97 (+++)
B	1:1:0	999.61	118.70	0.68 (+++)
HA1	3:1:1	917.23	102.50	0.39 (+++)
HA2	3:1:2	804.27	97.25	1.46 (+++)
HA3	3:1:3	770.82	113.30	1.55 (+++)
HB1	1:1:1	1020.45	124.80	0.10 (+++)
HB2	1:1:2	797.92	122.70	0.58 (+++)
HB3	1:1:3	404.79	102.70	1.46 (+++)

^1^ Values of hemolysis < 5% mean high hemocompatibility (+++) of material [19].

## Appendix A

SBF was prepared according to a recipe described elsewhere [20]. Briefly, powder reagents were dissolved in deionized water. The concentrations of several ions are shown in Table A1. Deionized water in the amount of 350 mL in a plastic beaker was heated to 36.5 ± 1.5 °C under continuous stirring. Reagents 1 to 8 were added successively in a given order. In case the total volume of the solution was under 450 mL, deionized water was added up to 450 mL volume in total. The pH of the solution before adding Tris had to be 2.0 ± 1. Tris was added into the solution little by little and pH was measured again and then HCl was added at temperature 36.5 °C until the pH was 7.40.

**Table A1:** Order, amount, and formula of reagents for preparing 500 mL of SBF.

Order	Reagent	Amount
1	NaCl	4.018 g
2	NaHCO_3_	0.178 g
3	KCl	0.114 g
4	K_2_HPO_4_ 3H_2_O	0.116 g
5	MgCl_2_ 6H_2_O	0.159 g
6	1M HCl	19.5 mL
7	CaCl_2_	0.146 g
8	Na_2_SO_4_	0.036 g
9	Tris	3.059 g
10	1M HCl	1.84 mL
